# Influence of graphene oxide on metal-insulator-semiconductor tunneling diodes

**DOI:** 10.1186/1556-276X-7-343

**Published:** 2012-06-26

**Authors:** Chu-Hsuan Lin, Wei-Ting Yeh, Chun-Hui Chan, Chun-Chieh Lin

**Affiliations:** 1 Department of Opto-Electronic Engineering, National Dong Hwa University, Hualien, 97401, Taiwan, Republic of China; 2 Department of Electrical Engineering, National Dong Hwa University, Hualien, 97401, Taiwan, Republic of China

**Keywords:** graphene oxide, MIS, tunneling diode, photodetector

## Abstract

In recent years, graphene studies have increased rapidly. Graphene oxide, which is an intermediate product to form graphene, is insulating, and it should be thermally reduced to be electrically conductive. We herein describe an attempt to make use of the insulating properties of graphene oxide. The graphene oxide layers are deposited onto Si substrates, and a metal-insulator-semiconductor tunneling structure is formed and its optoelectronic properties are studied. The accumulation dark current and inversion photocurrent of the graphene oxide device are superior to the control device. The introduction of graphene oxide improves the rectifying characteristic of the diode and enhances its responsivity as a photodetector. At 2 V, the photo-to-dark current ratio of the graphene oxide device is 24, larger than the value of 15 measured in the control device.

## Background

Metal-oxide-semiconductor field-effect transistors (MOSFETs) are frequently used in daily life. Strictly speaking, it is more precise to name such structures as metal-insulator-semiconductor field-effect transistors (MISFETs) since oxides represent only one class of the various insulators used today. To maximize the packing density and increase the operation speed of integrated circuits, transistor size must be reduced. The insulator thickness decreases as the dimension of a transistor becomes smaller, resulting in a significant gate tunneling current [1]. Such a tunneling current in the vertical direction (from metal to semiconductor or from semiconductor to metal) of a metal-insulator-semiconductor (MIS) structure has been employed in a number of applications, such as microwave devices [2], flash memory devices [3], solar cells [4], and photodetectors [5]. For photodetectors, the thickness of the insulator layer in the MIS tunneling diode is critical. For example, if the insulator layer is too thick, only limited tunneling can occur, leading to a small responsivity. On the contrary, if the insulator layer is too thin, Fermi level pinning may degrade the device current-voltage (IV) characteristics from rectifying behavior to ohmic behavior (or vice versa) [6,7]. In addition to the thickness dependence, insulator composition also affects the IV characteristics of MIS devices. In this letter, we describe the effect with graphene oxide introduced in the insulator layer in MIS tunneling diodes. Graphene studies increased rapidly following the report of Novoselov et al. in 2004 describing graphene obtained from repeated peeling of graphite [8]. Many methods to prepare graphene have since been reported [9-11]. One method involves chemical oxidation of graphite followed by exfoliation of graphene oxide (GO). These GO layers are insulating and could be thermally reduced to be electrically conductive [12]. Nevertheless, the insulating property of GO may be utilized to certain applications. Each GO layer consists of the pure two-dimensional honeycomb lattice bearing functional groups, and the two-dimensional structure is not present in commonly employed insulators such as amorphous SiO_2_. Therefore, the influence of the GO layer in the MIS structure will be very interesting. In this study, we demonstrate MIS tunneling diodes with GO in the insulator layer. We deposited the GO layers onto the Si substrates and evaluated these structures based on the IV characteristics. We highlight the simple fabrication process employed to achieve high-performance GO-based MIS devices.

## Methods

**Figure 1:**
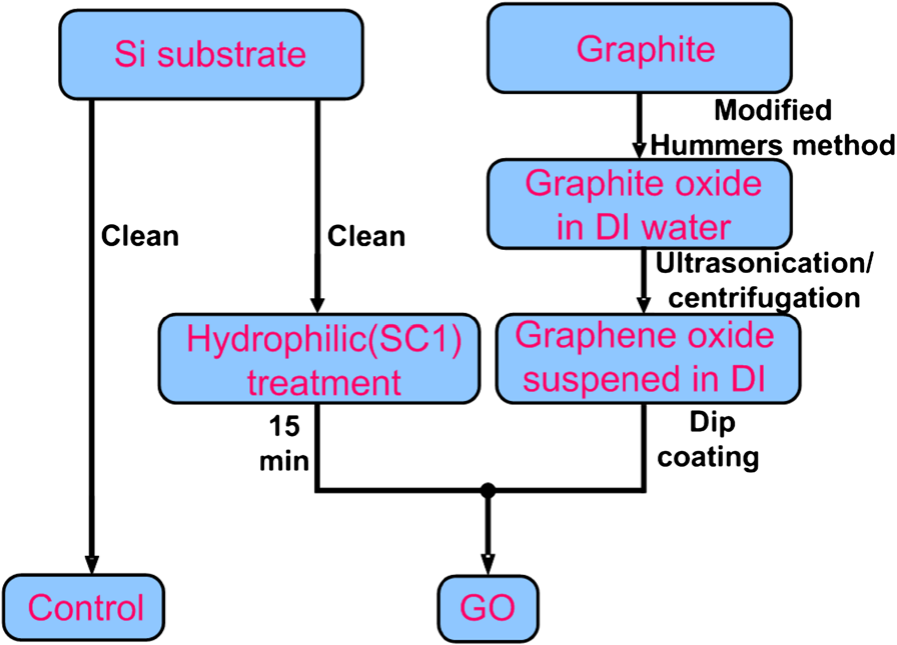
** Process flow to deposit graphene oxide on the Si substrate.** After ultrasonication and centrifugation, small GO sheets can be suspended in DI water. The hydrophilic treatment of the Si surface promotes GO sheet deposition on Si.

The schematic process used to prepare graphene oxide on Si substrates is shown in Figure 1, and the details are given below.

### Graphite oxidation (modified Hummers method)

Graphite oxide was prepared using the modified Hummers method [13,14]. First, H_2_SO_4_ (120 mL, 98%) was placed in an ice bath at < 5°C. Then, graphite powder (5 g) and KMnO_4_ (15 g) was slowly added and stirred in H_2_SO_4_ for 2 h. The temperature was controlled so as not to exceed 35°C. Deionized (DI) water (250 mL) was then slowly added to dilute the solution, which was then stirred for 2 h. DI water (700 mL) and H_2_O_2_ (20 mL) were then added and stirred for 12 h. The solution was filtered and the slurry on the filter paper was washed with HCl (3%) to remove inorganic impurities. The slurry was then washed with DI water (1 L) to remove the residual acid. Finally, the slurry was dried to yield graphite oxide.

### GO suspension

Graphite oxide powder (30 mg) was added to DI water (20 mL). After stirring for 10 min, ultrasonication was performed for 2 h to exfoliate the GO sheets from the multilayer flakes. The solution was then centrifuged at 5,000 rpm for 15 min, and the supernatant was collected. Due to the polar oxygen-containing functional groups, GO sheets were well suspended in the supernatant [15], which was subsequently used for dip-coating.

### Treatment on Si and GO dip-coating

In this study, 1- to 10-Ω cm p-type Si with native oxide was used as the substrates. The substrate was first cleaned and made hydrophilic by treatment with SC1 solution (NH_4_OH/H_2_O_2_/H_2_O = 1:2:8) for 15 min. The SC1 treatment rendered the surface hydrophilic by attaching polar hydroxyl groups as described in [16]. The GO sheets were then deposited on substrates by dip-coating. The GO deposition was driven by the van der Waals interaction between the hydrophilic substrate and the polar oxygen-containing groups of the GO. Following dip-coating for 40 min, the dried substrate was covered with multilayers of GO sheets. Each single GO sheet might be approximately 1 nm thick as described in [12]. However, the method of chemical exfoliation resulted in multiple-sheet deposition. From the profile of the atomic force microscopy (AFM) image, the thickness of the GO film in the GO device was estimated to be approximately 10 nm.

### Device fabrication

A 100-nm-thick Al gate with a circular area of 5 × 10^−4^ cm^2^ defined by a shadow mask was sputtered onto the GO-deposited side of the Si substrate. Al was also large-scale sputtered onto the back side of the Si substrate to form an ohmic contact. In order to identify the influence of the GO layer on the device characteristics, we also prepared and evaluated a control device without a GO film. White-light LEDs operating at a power density of 0.5 mW/cm^2^ were the illumination source used for photocurrent measurements.

## Results and discussion

**Figure 2:**
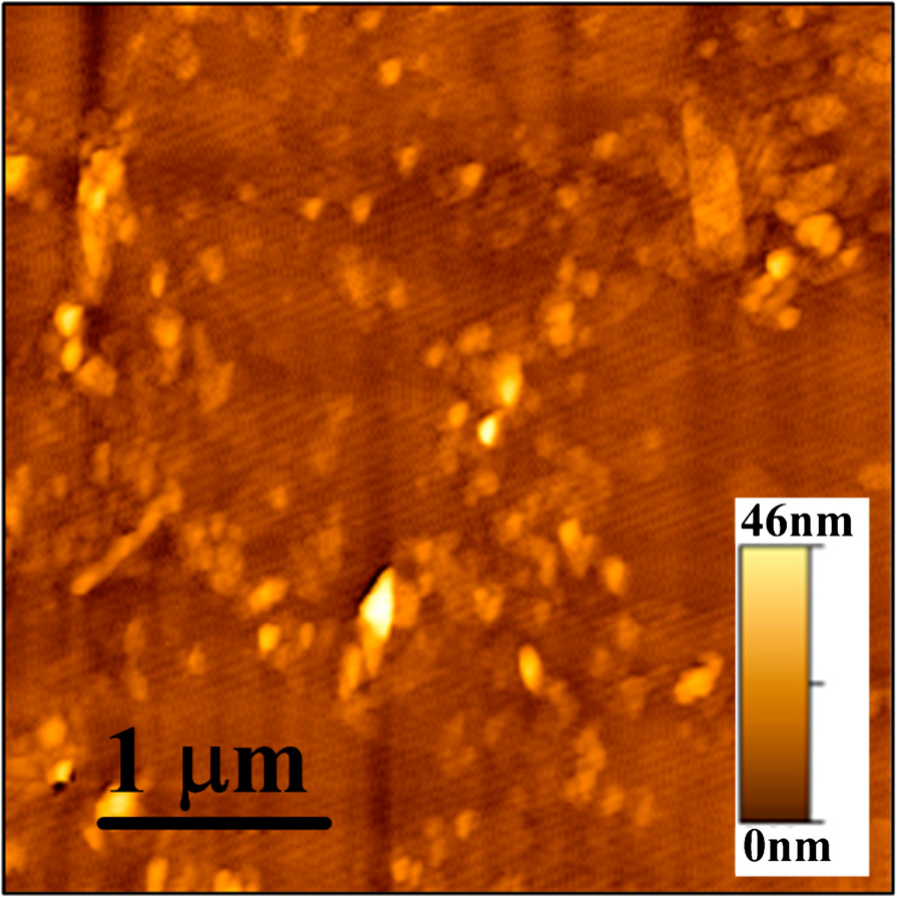
** AFM image of GO on the Si substrate.** The sample seems to be only partially covered with GO flakes.

**Figure 3:**
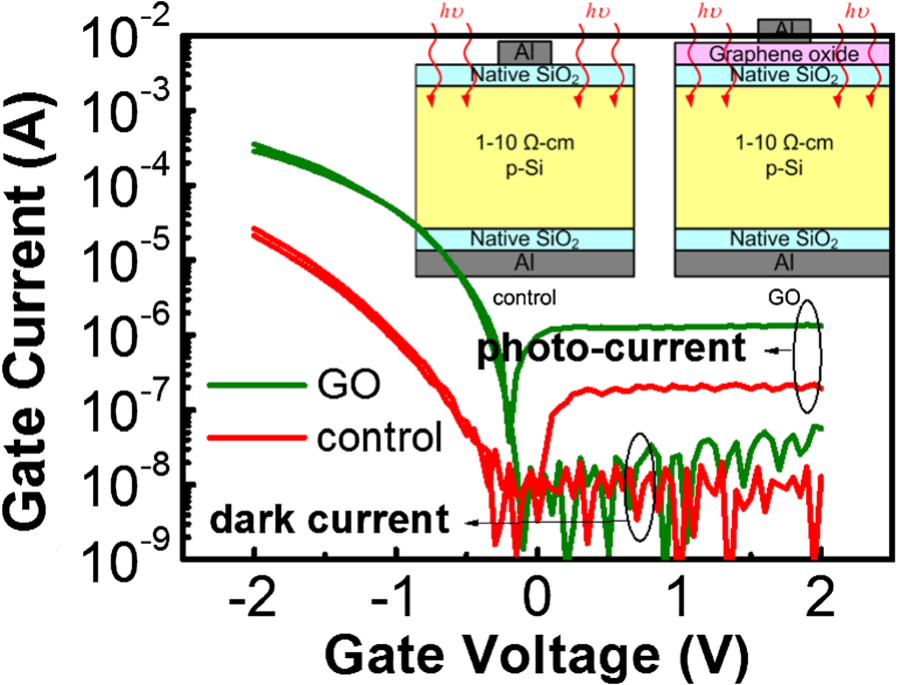
** Dark and photo-currents versus voltage characteristics of GO and control devices.** The inset shows the structures of GO and control devices. The accumulation (negative bias) dark current and inversion (positive bias) photocurrent of the GO device are superior to the control device.

**Figure 4:**
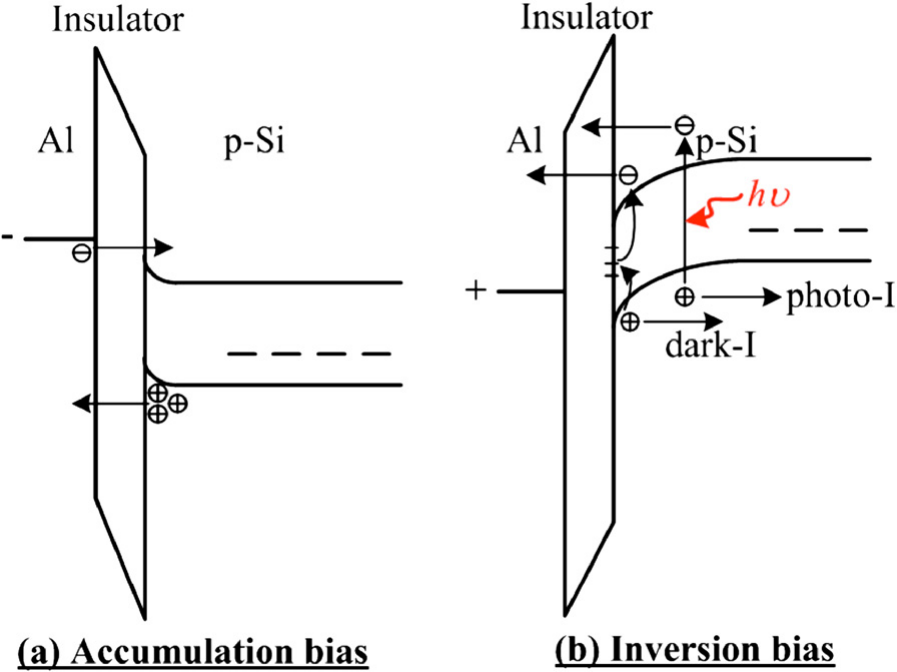
** Band diagrams of MIS tunneling diodes under (a) accumulation bias and (b) inversion bias.** Negative and positive biases applied to the top Al gate correspond to the accumulation and inversion operation biases for the MIS tunneling diodes on a p-type semiconductor.

We performed AFM to investigate the morphology of the GO sheets on the Si substrate (Figure 2). The root mean square roughness was 3.1 nm. It seemed that only the partial area was covered with GO flakes. The dark and photo-currents versus voltage characteristics of GO and control devices are shown in Figure 3, and the structures of GO and control devices are shown in the inset of Figure 3. The basic operation principle of an MIS photodetector can be found in [5]. Details regarding the conduction mechanisms in MIS tunneling diodes under an accumulation bias have been discussed in [17], and mechanisms under an inversion bias have been discussed in [18,19]. The tunnel diode is broadly used in low-power microwave applications. However, the MIS tunnel structure is also promising for use as a photodetector. As reviewed in [5], MIS tunneling diodes can be used to detect ultraviolet, visible, and infrared light. Photodetectors based on the MIS structure could own a smaller dark current than metal-semiconductor-metal photodetectors. The fabrication process is also simpler than that of pn photodetectors. Figure 4a,b shows the band diagrams under the accumulation bias and the inversion bias, respectively. Negative and positive biases applied to the top Al gate correspond to the accumulation and inversion operation biases, respectively, for the MIS tunneling diodes on a p-type semiconductor. At accumulation (negative) biases, holes are accumulated at the insulator/semiconductor interface and tunnel to the top gate. In addition, the Fermi level of the top gate continuously rises above the conduction band edge of the semiconductor. Hence, electrons can also tunnel from the top gate to the semiconductor (Figure 4a). The magnitude of the accumulation current is therefore related to the magnitude of the applied bias voltage (Figure 3). The magnitude of the inversion current is less dependent on the magnitude of the bias voltage. It is because that the inversion dark current is mainly contributed by thermally generated electron-hole pairs at the insulator/semiconductor interface and bulk traps in the depletion region. When the devices are irradiated, extra photo-generated electron-hole pairs result in a photocurrent (Figure 4b).

**Figure 5:**
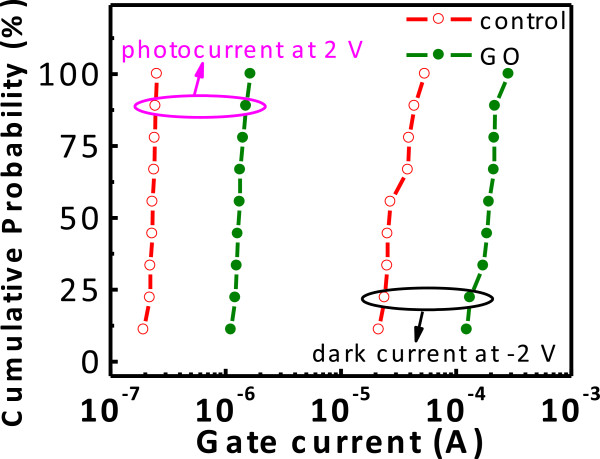
** Current cumulative probability plot of the GO and control devices.** All GO diodes exhibit larger currents than the control diodes whether for the accumulation dark currents or inversion photocurrents.

From the IV measurements, it is evident that the dark current of the GO device at the accumulation bias (for example, at −2 V) is larger than that of the control device. Since the magnitudes of the inversion (for example, at 2 V) dark currents of both devices are similar, the GO device has improved rectifying characteristics. At the inversion bias, the photocurrent of the GO device is also larger than that of the control device, i.e., at 2 V, the photocurrent of the GO MIS tunneling diode is 1.34 × 10^−6^ A, while that of the control diode is 1.94 × 10^−7^ A. This shows that the use of GO can increase the responsivity of MIS photodetectors. To show the characteristic variation within different devices, the cumulative probability plot of the collected data is shown in Figure 5. There are nine samples used both for the GO and control samples. In the cumulative probability plot, the formula of *i*/*n* is used for the cumulative probability calculation of the *y*-axis (in percentage), where *i* is the rank number and *n* is the number of data points. The presence of GO layers can indeed increase the accumulation (−2 V) dark currents and inversion (2 V) photocurrents measured in MIS devices.

At 2 V, the photo-to-dark current ratio of the GO device is 24 compared to the value of 15 obtained for the control device. The photocurrent of the GO device is much larger than that of the control device due to the introduction of GO. Meanwhile, the dark current of the GO device is only slightly increased. Overall, the GO device can achieve a better photo-to-dark current ratio.

In [18], the photocurrent density of an MIS tunneling diode is approximately 0.03 mA/cm^2^ under 0.71-mW/cm^2^ illumination. The photocurrent density of our GO MIS tunneling diode is approximately 3 mA/cm^2^ under 0.5-mW/cm^2^ illumination; therefore, a much higher responsivity could be achieved via GO incorporation. Although the smaller dark current of the diode reported in [18] would result in a better photo-to-dark current ratio, the photo-to-dark current ratio of our GO device could increase if the GO is deposited on a high-quality thermal oxide as opposed to the native oxide.

**Figure 6:**
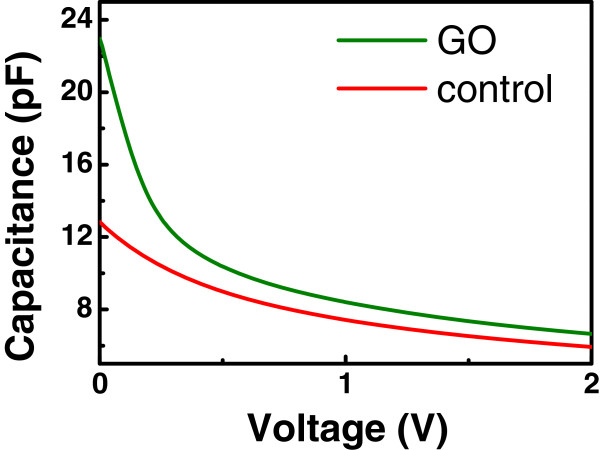
** Capacitance-voltage relation of the GO and control devices.** The negative-bias data could not be obtained due to the large accumulation currents of our devices. The shift of the GO curve as compared to the control curve may be due to the negative interface charge (or oxide charge) of GO.

The beneficial influence of GO may be attributed to its negative interface charge (or oxide charge). Al_2_O_3_ has been previously used to passivate the surface of p-Si due to the negative charge at its interface, and the capacitance-voltage curves shift toward positive voltage when more negative charge is present [20]. We have measured the capacitance-voltage relation of the GO and control samples. Although the negative-bias data could not be obtained due to the large accumulation currents of our devices, the positive-bias data show a positive shift similar to the Al_2_O_3_ case (Figure 6). If there is negative charge at the interface, holes prefer to accumulate at the surface of p-Si. Minority carriers, electrons, could be pushed away from the interface of p-Si, and it leads to a low surface recombination [20]. Low surface recombination could contribute to the high inversion photocurrent of the GO sample. On the other hand, the inherent hole accumulation present at the surface of p-Si of the GO sample also results in the larger accumulation currents observed at the negative bias.

Although thick GO flakes may prevent the tunneling of carriers, carriers can still flow through the native oxide that it is not covered with thick GO flakes for the GO sample. In other words, the GO sample is only partially covered with GO, and carriers can tunnel via the uncovered regions.

**Figure 7:**
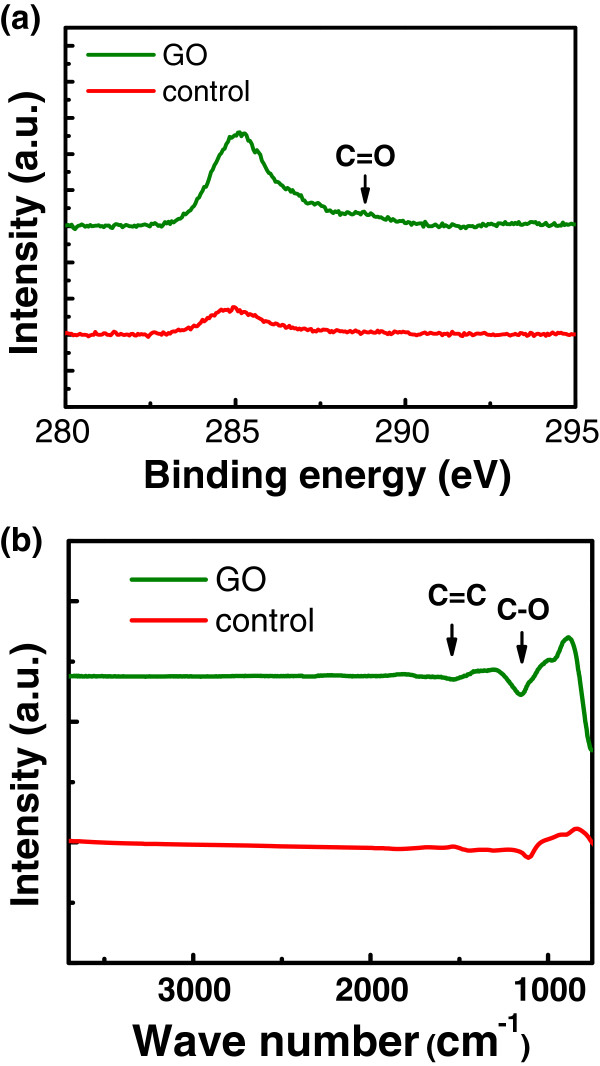
** XPS spectra (a) and reflective FTIR spectra (b) of GO and control samples.** The carbon-oxygen functional groups, such as C = O and C-O, are observed.

We used X-ray photoelectron spectroscopy (XPS) and reflective Fourier transform infrared spectroscopy (FTIR) to identify the chemical composition of the GO films. Figure 7a shows the carbon (C) 1*s* XPS spectra of GO and control samples. The peak at approximately 285 eV corresponds to C 1*s* peak of an *sp*^2^ C or *sp*^3^ C [21], and the small peak at approximately 289 eV corresponds to a carbonyl carbon (C = O) [22]. Since the control sample has been cleaned in acetone and methanol, a small C 1*s* peak at approximately 285 eV can be observed. Figure 7b shows the reflective FTIR spectra of GO and control samples. The GO spectrum is obtained with the Si substrate used in background measurement; hence, the final spectrum only reveals the reflection characteristics of the graphene-oxide film. In addition to the absorption due to C = C bonds, the absorption due to C-O bonds can also be observed. Similar results have been observed in [23]. The ‘carbon-oxygen’ functional groups are responsible for the insulating properties of GO. We attempted to perform Hall measurement to obtain the resistivity and mobility of the GO films; however, since the top layers were insulators instead of semiconductors, no reliable electrical data could be obtained.

## Conclusions

Graphene oxide was deposited on Si to form an Al/graphene oxide/native oxide/Si MIS tunneling diode. With GO insertion, the accumulation dark current and inversion photocurrent are greater than those measured in the control device. The photocurrent of the GO MIS tunneling diode is 1.34 × 10^−6^ A, while the photocurrent of the control diode is 1.94 × 10^−7^ A; thus, the GO-based device is a promising candidate for detector applications. Future work can involve optimization of GO coverage in MIS tunneling diodes.

## Competing interests

The authors declare that they have no competing interests.

## Authors’ contributions

CHL conceived the experimental methodology, modified the process flow, and prepared the manuscript. WTY and CHC performed the device fabrication and measurement. CCL assisted with the device fabrication and participated in the modification of the methodology. All authors read and approved the final manuscript.

## Authors’ information

CHL is an assistant professor in the Department of Opto-Electronic Engineering, National Dong Hwa University. WTY and CHC are currently working toward their master degree in the Department of Opto-Electronic Engineering, National Dong Hwa University. CCL is an assistant professor in the Department of Electrical Engineering in the same school.
